# Comparative study of acute kidney injury caused by isotonic and low-osmolar iodinated contrast media in patients with renal insufficiency

**DOI:** 10.1515/med-2026-1472

**Published:** 2026-08-31

**Authors:** Ya Li, Yan Wang, Mengqi Hao, Hua Ling, Ling Hu, Jian Liu, Han Zhou, Zhengkai Zhao

**Affiliations:** Department of Radiology, Department of Oncology and Department of Gastroenterology, Chengdu Third People’s Hospital, Chengdu, China

**Keywords:** iodine, contrast media, renal insufficiency, acute kidney injury

## Abstract

**Objectives:**

To assess the incidence and severity of contrast-induced acute kidney injury(PC-AKI) cause by isotonic and low-osmolar iodinated contrast media of patients with renal insufficiency undergoing intravenous administration.

**Methods:**

We collected the patients diagnosed with confirmed renal insufficiency after January 1, 2022, and they were scheduled for enhanced CT examination. Then these patients were randomly divided into the isotonic contrast agent Iodixanol group, the hypertonic contrast agent Ioversol group, Iohexol group, Iomeprol group and Iopromide group. After hospitalization, these patients were routinely checked for renal function and calculated glomerular filtration rate. Finally, these patients underwent renal function examination again within 48–72 h and the glomerular filtration rates were calculated.

**Results:**

There were statistical differences in age and cystatin C among the groups. However, it was no significant difference in serum creatinine, glomerular filtration rate before CT enhancement and serum creatinine, glomerular filtration rate, uric acid and urea after CT enhancement in each group. For patients with renal insufficiency, the incidence of PC-AKI caused by isotonic contrast agents and multiple hypertonic contrast agents was 0–4.3 %, and there was no statistical difference in the incidence of PC-AKI among the groups.

**Conclusions:**

Isotonic contrast agent and hypertonic contrast agent all have good renal safety in people with renal insufficiency.

## Introduction

Contrast-induced acute kidney injury (CI-AKI), formerly known as contrast-induced nephropathy, refers to acute renal dysfunction occurring after intravascular administration of iodinated contrast agents [1]. The risk of CI-AKI is directly proportional to the severity of renal insufficiency. With the increasing use of contrast-enhanced CT technology, the widespread utilization of iodinated contrast agents has led to a rising incidence of CI-AKI, imposing a significant socioeconomic burden. Consequently, the importance of preventing and managing CI-AKI has become increasingly evident [2].

Previously, CI-AKI was diagnosed based on an absolute increase in serum creatinine of ≥44.2 μmol/L (0.5 mg/dL) or a relative increase of ≥25 % from baseline, referred to as the PC-AKI 25 % criteria [3]. However, recent modifications to diagnostic criteria have revised the timing and standards for serum creatinine measurement, including those by the European Society of Urogenital Radiology (ESUR) guidelines in 2018 and the consensus statement on intravenous iodinated contrast media use in kidney disease jointly released by the American College of Radiology (ACR) and the National Kidney Foundation (NKF) in 2020. The new definition of CI-AKI is an absolute increase in serum creatinine of ≥26.5 μmol/L (0.3 mg/dL) or a relative increase of ≥50 % from baseline within 48–72 h after contrast administration [2], 4].

Currently, there is limited research investigating CI-AKI following intravenous administration of isotonic and low-osmolar iodinated contrast media based on the new criteria for acute kidney injury diagnosis. Hence, this prospective study aims to evaluate the incidence and severity of CI-AKI in patients with renal insufficiency after administration of isotonic and low-osmolar iodinated contrast media intravenously, utilizing the updated diagnostic criteria for acute kidney injury.

## Materials and methods

### Study subjects

Patients admitted to the Third People’s Hospital of Chengdu City after January 1, 2022, with confirmed renal insufficiency and scheduled for contrast-enhanced CT examination, were included in this study. Then they were randomly assigned to receive either Iodixanol, Ioversol, Iohexol, Iomeprol, or Iopromide. Iodixanol was used as an iso-osmolar contrast agent, while the others were used as hypertonic contrast agent.

Upon admission, routine renal function tests were performed, and glomerular filtration rate (GFR) was calculated. General information and medical history were collected from the patients. Within 48–72 h after administration of the contrast agent, renal function tests were repeated, and serum creatinine, uric acid, urea, and cystatin C levels were measured. GFR was recalculated based on these parameters.

Inclusion criteria were as follows: (1) aged 18 years or older, (2) confirmed renal insufficiency before CT enhancement (30 mL/(min·1.73 m2) < eGFR<90 mL/(min·1.73 m2)), (3) undergone at least one contrast agent examination, and (4) signed informed consent.

Exclusion criteria were: (1) hyperthyroidism not reaching a severe stage, (2) history of iodine contrast agent allergy, (3) interval between adjacent contrast agent uses less than 72 h, (4) use of nephrotoxic drugs from 24 h before the first contrast agent examination to 72 h after the examination, (5) receipt of more than two different iodine contrast agent examinations during hospitalization, (6) Nephrotoxic drugs included nonsteroidal anti-inflammatory drugs (NSAIDs), aminoglycoside antibiotics, vancomycin, amphotericin B, and chemotherapeutic agents, (7) Patients with active malignancy were excluded from the study, (8) other circumstances such as pregnancy or lactation.

A total of 211 patients with renal insufficiency were enrolled in this study, including 46 in the Iodixanol group, 45 in the Ioversol group, 44 in the Iohexol group, 44 in the Iomeprol group, and 32 in the Iopromide group. All patients underwent a single contrast-enhanced CT examination during the study period.

Baseline demographic and clinical data were collected, including age, sex, body mass index, smoking and alcohol history, and comorbidities such as diabetes and hypertension.Serum creatinine measurements were performed using standardized laboratory methods in accordance with hospital protocols.Cystatin C was measured only once within 48–72 h after contrast administration. This study has been reviewed by the Ethics Committee of Chengdu Third People’s Hospital, and the ethics project number is HX-IRB-AF-05-V3.0. All subjects signed informed consent in writing.Informed consent was obtained from all individuals included in this study, or their legal guardians or wards.

### Contrast agent

All contrast agents used in this study were non-ionic iodinated contrast media administered via intravenous injection. Iodixanol was used as an iso-osmolar contrast agent, with an osmolality similar to that of plasma (approximately 290 mOsm/kg). In contrast, ioversol, iohexol, iomeprol, and iopromide were classified as low-osmolar contrast agents, with osmolality ranging from 600 to 800 mOsm/kg.The selection of contrast agents was based on clinical practice, and all agents were administered according to standard dosing protocols. The type and dosage of contrast media were determined by the attending radiologist based on patient characteristics and examination requirements. For all contrast agents, the standard dose was 1.5 mL/kg of body weight, with maximum total doses not exceeding 150 mL for iodixanol and 120 mL for low-osmolar contrast agents. Doses were adjusted as necessary according to the patient’s renal function, body weight, and clinical condition to ensure safety. All injections were performed intravenously using a power injector at the recommended flow rates.

### Relevant definitions

The glomerular filtration rate (GFR) is calculated using the simplified Modification of Diet in Renal Disease (MDRD) equation: eGFR=186 × Scr^−1.154^ × Age^−0.203^ (female × 0.742) [unit: mL/(min·1.73 m^2^)].

PC-AKI classification:

Grade I: Serum creatinine elevation ≥26.5 μmol/L (0.3 mg/dL) or 1.5–1.9 times above baseline after contrast agent use.

Grade II: Serum creatinine elevation 2.0–2.9 times above baseline after contrast agent use.

Grade III: Any of the following criteria:

(1) Serum creatinine elevation ≥533.6 μmol/L (4.0 mg/dL) or 3 times above the upper limit after contrast agent use.
(2) Requirement of renal replacement therapy.

### Statistical analysis

Data analysis was conducted using SPSS 21.0. The comparison of categorical data was performed using the Kruskal-Wallis test. For continuous data, mean ± standard deviation (X̅±s) was used, and intergroup comparisons were conducted using one-way analysis of variance (ANOVA). Two-group comparisons were analyzed using independent samples t-test. p<0.05 was considered statistically significant.

## Results

### The comparison of general information among different groups

It has presented the comparison of clinical data among groups in Table 1. Statistical differences were observed between the Iohexol and Iomeprol groups for gender index. Regarding diabetes indicators, statistically significant differences were noted between the Ioversol and Iohexol groups as compared to the Iodixanol group. Smoking history indicators showed statistical differences between the Iodixanol and Iohexol groups. Hypertension indicators exhibited statistical differences between the Iomeprol and Iopromide groups. Age indicators demonstrated statistical differences between the Iodixanol group and theIoversol, Iohexol, and Iopromidegroups, as well as between the Iomeprol group and the Ioversol, Iohexol, and Iopromide groups. Height indicators revealed statistical differences between the Iohexol and Iopromide groups.

**Table 1:** The comparison of clinical data among different groups.

Category	Iodixanol group (n=46)	Ioversol group (n=45)	Iohexol group (n=44)	Iomeprol group (n=44)	Iopromide group (n=32)	F/X^2^ value	p-Value
Gender, male/female	26/20	26/19	21/23	21/23	17/15	4.2	0.37
Smoking history, yes/no	13/33	11/34	4/40	4/40	7/25	5.6	0.24
Alcohol consumption history, yes/no	11/35	11/34	4/40	4/40	7/25	7.2	0.13
Diabetes history, yes/no	14/32	3/42	4/40	4/40	6/26	11.6	0.02
Hypertension history, yes/no	11/35	11/34	10/34	10/34	3/29	4.2	0.38
Age, x ± s	77.2 ± 9.9	69.4 ± 11.5	67.1 ± 8.9	67.1 ± 8.9	64.6 ± 11.4	9.5	<0.01
Height/cm, x ± s	161.1 ± 8.2	161.1 ± 7.6	158.9 ± 10.6	158.9 ± 10.0	163.3 ± 7.0	1.2	0.30
Weight/Kg, x ± s	59.4 ± 10.4	60.3 ± 9.9	57.3 ± 11.8	61.6 ± 11.3	60.00 ± 11.3	0.9	0.46

F represents the F-statistic from one-way analysis of variance (ANOVA) for continuous variables, whereas X^2^ denotes the Chi-square statistic for categorical variables.

### The comparison of indicators in each group pre- and post-enhancement CT examination

The comparison of pre- and post-enhancement CT examination indicators among groups was shown in Table 2 and 3. In comparisons between groups, there were statistically significant differences in the indicators of glomerular filtration rate (GFR) before examination among the Iodixanol group, Iohexol group, and Iopromide group. For urea indicators, there were statistically significant differences between the Iomeprol group and the Iodixanol group, Iohexol group, and Iopromide group. Regarding cystatin C indicators, there were statistically significant differences between the Iodixanol group and the Iopromide group, and between the Iomeprol group and the Ioversol group, Iohexol group, and Iopromide group. There were no statistically significant differences in other indicators among groups. The number of cases of contrast-induced acute kidney injury (CI-AKI) in the Iodixanol group, Ioversol group, Iohexol group, Iomeprol group, and Iopromide group were 2, 1, 0, 1, and 1 respectively, with incidence rates of 4.3 %, 2.2 %, 0 %, 2.3 %, and 3.1 %, respectively. There were no statistically significant differences in comparisons between groups. The overall incidence rate of CI-AKI in this study was 2.4 %, with one case in the Iodixanol group classified as PC-AKI II and the remainder classified as PC-AKI I.

**Table 2:** The comparison of pre- and post-enhancement CT examination indicators among groups.

Indicator	Iodixanol group (n=46)	Ioversol group (n=45)	Iohexol group (n=44)	Iomeprol group (n=44)	Iopromide group (n=32)	F value	p-Value
Pre-serum creatinine, μmol/L	82.0 ± 23.2	87.4 ± 26.9	78.6 ± 22.6	87.4 ± 24.9	79.0 ± 18.0	1.4	0.24
Pre-glomerular filtration rate (mL/min/1.73 m^2^)	67.8 ± 15.9	71.9 ± 16.9	74.4 ± 14.1	72.7 ± 16.0	75.5 ± 12.6	1.6	0.19
Post-serum creatinine, μmol/L	77.7 ± 20.8	85.5 ± 30.5	78.2 ± 25.2	82.0 ± 23.8	76.5 ± 21.1	0.9	0.44
Post-glomerular filtration rate (mL/min/1.73 m^2^)	72.2 ± 20.8	73.5 ± 18.9	77.0 ± 15.2	76.0 ± 17.4	78.7 ± 15.1	0.9	0.46
Uric acid, μmol/L	276.2 ± 106.6	299.9 ± 91.2	300.2 ± 88.5	315.5 ± 108.2	307.8 ± 103.8	1.0	0.44
Urea, mmol/L	5.3 ± 2.7	5.7 ± 3.5	5.0 ± 1.8	6.6 ± 4.2	4.7 ± 1.8	2.4	0.05
Cystatin C, mmol/L	1.2 ± 0.3	1.1 ± 0.4	1.1 ± 0.2	1.3 ± 0.4	1.2 ± 0.2	3.8	<0.01

**Table 3:** Incidence of PC-AKI by grade in each group.

Group	n	PC-AKI I	PC-AKI II	PC-AKI III
Iodixanol	46	1 (2.2 %)	1 (2.2 %)	0 (0 %)
Ioversol	45	1 (2.2 %)	0 (0 %)	0 (0 %)
Iohexol	44	0 (0 %)	0 (0 %)	0 (0 %)
Iomeprol	44	1 (2.3 %)	0 (0 %)	0 (0 %)
Iopromide	32	1 (3.1 %)	0 (0 %)	0 (0 %)

## Discussion

Enhanced CT and angiography techniques have become increasingly prevalent in recent years, leading to a rising frequency of iodine contrast agent use. The safe, standardized, and rational application of iodine contrast agents has garnered significant attention. Post-contrast acute kidney injury (PC-AKI) is a common adverse reaction following the use of iodine contrast agents and ranks as the third leading cause of iatrogenic acute kidney injury, accounting for 9.1 % of cases [5]. Previous studies have identified three main reasons for PC-AKI occurrence: Firstly, contrast agents induce renal vasoconstriction, leading to a decrease in renal glomerular plasma flow and shortening the effective length for glomerular capillary filtration, resulting in decreased eGFR. Secondly, contrast agents cause cytotoxic damage to tubular epithelial cells, altering filtration membrane permeability and further decreasing eGFR. Thirdly, contrast agents increase plasma viscosity, reducing renal glomerular capillary flow and altering effective filtration pressure, consequently lowering eGFR [3], 6]. The primary influencing factors for PC-AKI are the osmolality and viscosity of iodine contrast agents. Isotonic contrast agent offers the advantage of lower osmolality, but its higher viscosity compared to hypertonic contrast agent may pose greater renal toxicity risks [4].

For patients with renal insufficiency, there has been a historical concern about increased risk of post-contrast acute kidney injury (PC-AKI), often leading to the avoidance or delay of iodine contrast agent use, which can result in misdiagnosis or delayed treatment. Therefore, the safety of iodine contrast agents in clinical practice has been a significant focus of attention.The 2020 consensus from ACR (American College of Radiology) and NKF (National Kidney Foundation) indicates that the risk of acute kidney injury following contrast agent use increases with the severity of chronic renal insufficiency(CKD). The risk percentages post-contrast agent use are approximately 5 % for eGFR≥60 mL/(min·1.73 m^2^), 10 % for eGFR 45–59, 15 % for eGFR 30–44, and 30 % for eGFR<30 [2]. Risk factors for PC-AKI also include nephrotoxic medications, advanced age, hypotension, diabetes mellitus, anemia, chronic heart failure, higher contrast agent doses, and shorter intervals between contrast agent administrations [7], [8], [9]. In this study, Iodixanol group had a higher incidence of PC-AKI compared to other groups, possibly due to a higher prevalence of diabetes mellitus and older age within Iodixanol group. The overall incidence of PC-AKI in the general population is typically between 0.6 and 2.3 % based on the PC-AKI 25 % criteria [10]. However, meta-analyses have shown rates between 4.96 % and 6.4 % [11], 12]. This study found an overall incidence rate of PC-AKI of 2.4 % for patients with renal insufficiency and using the PC-AKI 50 % criteria.

For the 25 % PC-AKI criterion, Yang et al. showed no statistically significant difference in the incidence of acute kidney injury after the use of iodinated contrast agents between patients with normal renal function and those with renal insufficiency by the meta-analysis [13]. Two other meta-analyses indicated that there was no statistically significant difference in renal safety between hypertonic contrast agent (excluding Iohexol and Iodixanol) and isotonic contrast agent [14], 15]. A systematic review and meta-analysis indicate that, in hemodynamically unstable patients, targeting a higher mean arterial pressure (MAP) does not significantly reduce the incidence of AKI compared with maintaining normotension. Evidence from multiple randomized controlled trials also shows that iso-osmolar and low-osmolar contrast agents do not differ consistently in their risk of post-contrast AKI, and any modest differences observed in subgroup analyses are neither statistically nor clinically significant. [16]. This study found no significant differences among groups in pre-examination serum creatinine and glomerular filtration rate, post-examination serum creatinine and glomerular filtration rate, uric acid, or urea. It should be noted that cystatin C levels may be influenced by various factors independent of renal function, including corticosteroid use, thyroid dysfunction, inflammation, and malignancy. These potential confounding factors were not fully controlled in this study, which may have affected the observed differences in cystatin C levels among groups.For patients with renal insufficiency and based on the 50 % PC-AKI criterion, the incidence of PC-AKI caused by isotonic contrast agent and various types of low-osmolar contrast agents ranged from 0 to 4.3 %, with no statistically significant differences in PC-AKI incidence among the groups. There was one case of PC-AKI grade 2 in the Iodixanol group, and the remainder were PC-AKI grade 1.The novelty of this study lies in the evaluation of PC-AKI incidence based on the updated 50 % diagnostic criteria in patients with renal insufficiency undergoing intravenous contrast administration. Additionally, multiple commonly used iso-osmolar and low-osmolar contrast agents were directly compared in a real-world clinical setting, providing practical evidence for their renal safety.

## Conclusions

In summary, for patients with renal insufficiency based on the 50 % PC-AKI criterion, the incidence of PC-AKI caused by isotonic and various hypertonic contrast agents ranges from 0 to 4.3 %, with no statistically significant differences among groups. This demonstrates that both isotonic contrast agent and hypertonic contrast agent maintain good renal safety profiles in populations with renal insufficiency.

## Study limitation

(1) The study focused on patients with stage 2–3 CKD. There was no comparative research conducted on individuals with normal kidney function or those in CKD stages 4–5.
(2) Only iso-osmolar contrast agent iodixanol and several types of low-osmolar contrast agents including iopromide, iohexol and iomeprol were studied. Other types of contrast agents were not included in the study.
(3) The study had a limited number of cases; further improvement in sample size and multicenter studies are needed.
(4) Although some baseline variables showed statistical differences among groups, no significant differences were observed in key renal function indicators before and after contrast administration.
(5) This study lacked follow-up renal function data for patients who developed PC-AKI, so recovery could not be evaluated. However, previous studies in stage 2–3 CKD indicate that most PC-AKI events are mild and reversible.
(6) A limitation of this study is the lack of detailed data on patients’ medications and comorbidities, which may affect PC-AKI risk.
